# Novel approach in the resection of oral maxillary tumours

**DOI:** 10.4317/medoral.27046

**Published:** 2025-03-23

**Authors:** Pablo Cea-Arestín, Arturo Bilbao-Alonso, Abel García-García, Carolina Menéndez-Lago

**Affiliations:** 1 ORCID ID: 0000-0002-0655-0412. Associate. Department of Surgery and Medical-Surgical Specialties, School of Medicine and Dentistry, University of Santiago de Compostela, Santiago de Compostela, Galicia, Spain; 2 ORCID ID: 0000-0002-3224-1007. MD, PhD, PhD, OMS. Maxillofacial Surgery Service, Santiago de Compostela Hospital Complex (CHUS), Galician Health Service (SERGAS). ZAGA Center A Coruña and Lugo, Galicia, Spain; 3 MD, PhD. Faculty of Medicine and Dentistry, University of Santiago de Compostela, Santiago de Compostela, Spain; Maxillofacial Surgery Service, Santiago de Compostela Hospital Complex (CHUS), Galician Health Service (SERGAS), Spain; 4 Private practice, Santiago de Compostela, Galicia, Spain

## Abstract

**Background:**

Oral cavity tumours represent a global oral health issue due to their prevalence and incidence, in this paper, we will explain the procedure for the removal of various tumours and the restoration of the patient's anatomy, function and aesthetics through a novel approach that rely on a temporal muscle flap for the reconstruction of soft tissues in the surgical area.

**Material and Methods:**

This study is based on an analysis of temporal flap technique data, collected at the public health hospital of Santiago de Compostela (CHUS), from 2015 to 2024.

**Results:**

The results are shown in Table 1, being the most relevant data the fact of having a series of 11 successful cases treated with temporal flap. The surgical technique involves tumor resection, preferably through an intraoral approach.

**Conclusions:**

This novel technique must be taken into account when solving this type of oncological cases with associated maxillectomy due to it’s better results in terms of reconstruction of tissues and anatomy, shorter execution time and possibility of offering immediate function to the patient, leading to less number of surgeries with their consequent lower morbidity and social-health and economic savings.

** Key words:**Zygomatic tumor, zygomatic tumour, zygomatic oncology implant.

## Introduction

Oral cavity tumours represent a global oral health issue due to their prevalence and incidence (1), with data showing that in 2022 the number of new cases detected reached 389,485 bringing a mortality rate of nearly 50% (2).

In this paper, we will explain the procedure for the removal of various tumours and the restoration of the patient's anatomy, function and aesthetics through a novel approach supported by the success of several performed cases.

After tumour removal, we will rely on a temporal muscle flap for the reconstruction of soft tissues in the surgical area along with the use of conventional dental, zygomatic and pterygoid implants, all of which have shown high success rates in such situations (3).

In particular, zygomatic implants have been proved especially useful, as one of their main indications is their use after maxillectomies due to oncologic causes (4-7).

## Material and Methods

This study is based on an analysis of temporal flap technique data, collected at the public health hospital of Santiago de Compostela (CHUS), from 2015 to 2024.

The data coding, processing and statistical analysis were conducted using Excel as the primary computational and mathematical tool.

All ethical, healthcare, legal, data management and administrative requirements were strictly adhered to and in accordance with current regulations.

## Results

The results are shown in Table 1, being the most relevant data the fact of having a serie of 11 successful cases.

A total of 11 patients were treated using this technique, temporal flap taken in 11 cases (100%), immediate implants were placed in 4 cases (36,36%) and the number of zygomatic implants positioned per case were 1 in 8 cases (72,73%) and 2 in 3 cases (27,27%).

The surgical technique involves tumor resection, preferably through an intraoral approach, in cases where this is not possible, a Weber-Ferguson approach will be used. After marking the resection area, the tooth in the premaxilla contiguous to the resective margin is extracted to allow the implant to be placed in the most medial location, based on findings from the finite element load distribution study (7-8). The osteotomy, usually up to the nasal septum to ensure the medial margin, is completed using piezoelectric instruments.

**Figure 1:**
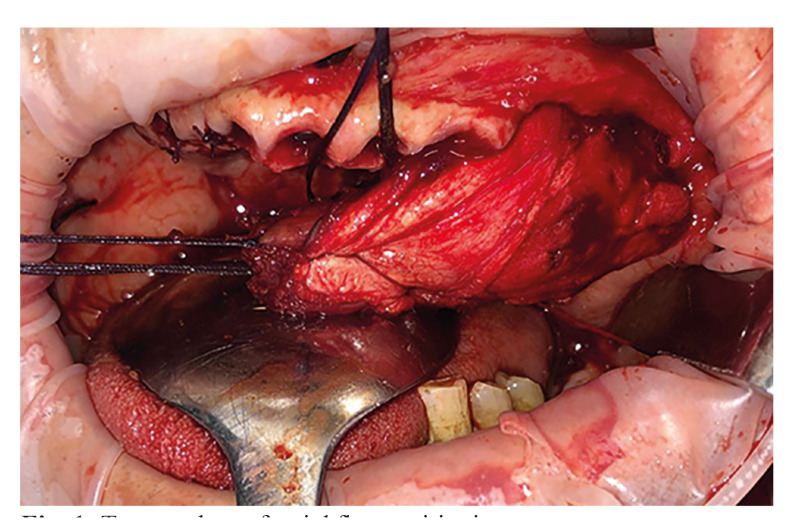
Temporal myofascial flap positioning.

To harvest the temporal muscle graft, we approach the temporal area itself following the hairline to avoid potential areas of alopecia and the dissection of the temporal fascia is then performed lifting it carefully to include the facial nerve to prevent damage. Once reaching the bony plane at the temporal fossa, zygomatic arch, and frontomalar region, the temporal myofascial flap is detached and passed under the zygomatic arch, if the passage is too narrow or the flap is too large, osteotomy followed by osteosynthesis may be required (Fig. 1). This procedure serves as an alternative to harvesting the flap from the arm (9), thus resulting in lower morbidity.

**Figure 2:**
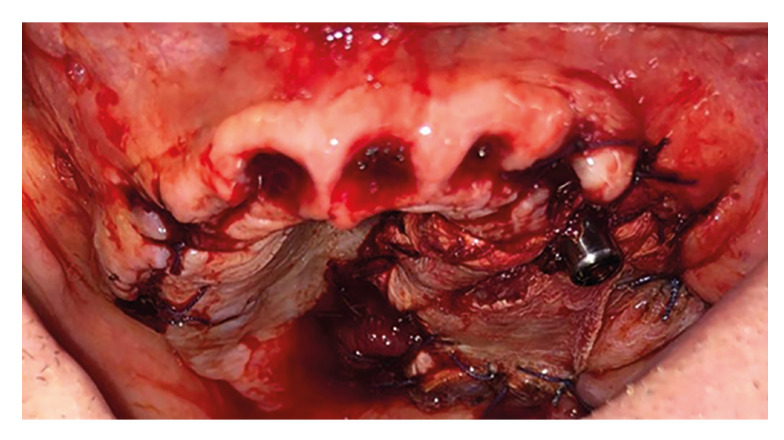
Zygomatic implant surrounded by temporal flap.

Once the flap is positioned in the oral cavity, the implants are placed, aiming to position at least one medial implant, one zygomatic implant, and, if oncologically feasible, a pterygoid implant (4,7), with the temporal flap ultimately positioned over the implants (Fig. 2).

## Discussion

The indications for this type of surgery include: patients without prior reconstruction following a maxillectomy, patients with soft tissue reconstruction without bone reconstruction or implants, and patients undergoing simultaneous resection, reconstruction, and implant placement. The effectiveness and predictability of this procedure have been proven, with high implant survival rates (10).

The next step in this technique would be to make it a guided process with a fully digital workflow for maximum precision, this is feasible since there are currently digital guided methods with slight variability that still allow for prosthesis placement in the same procedure (11).

## Conclusions

This novel technique must be taken into account when solving this type of oncological cases with associated maxillectomy due to it’s better results in terms of reconstruction of tissues and anatomy, shorter execution time and possibility of offering immediate function to the patient, leading to less number of surgeries with their consequent lower morbidity and social-health and economic savings.

## Figures and Tables

**Table 1:** Results of analysis of temporal flap technique data.

Patient	Year	Diagnosis	Location	Flap	II	IP	ZI	Brand	Relapse
1	2015	OSCC	Right	Temporal	No	Yes	1	NB	No
2	2020	OSCC	Central	Temporal	No	Yes	2	SI	Yes
3	2021	AFL	Left	Temporal	No	Yes	1	SI	No
4	2021	CYL	Left	Temporal	No	Yes	1	SI	Yes
5	2021	ENB	Right	Temporal	No	No	2	SI	No
6	2023	CYL	Left	Temporal	Yes	No	1	SI	No
7	2023	CR	Bilateral	Temporal	No	Yes	2	SI	No
8	2023	AC	Right	Temporal	Yes	No	1	SI	No
9	2024	OSCC	Left	Temporal	Yes	No	1	SI	No
10	2024	OSCC	Left	Temporal	Yes	No	1	SI	No
11	2024	SC	Right	Temporal	No	No	1	SI	No

II: Immediate implant; IP: Immediate prosthesis; ZI: Zygomatic implants; OSCC: Oral Squamous Cell Carcinoma; AFL: Angiofibrolipoma; CYL: Cylindroma; ENB: Estesioneuroblastoma; CR: Cavum Radionecrosis; AC: Adenocarcinoma; SC: Sarcoma; NB: Nobel Biocare; SI: Southern Implants.
